# Factors affecting soil microbial biomass and functional diversity with the application of organic amendments in three contrasting cropland soils during a field experiment

**DOI:** 10.1371/journal.pone.0203812

**Published:** 2018-09-13

**Authors:** Ling Li, Minggang Xu, Mohammad Eyakub Ali, Wenju Zhang, Yinghua Duan, Dongchu Li

**Affiliations:** 1 National Engineering Laboratory for Improving Quality of Arable Land, Institute of Agricultural Resources and Regional Planning, Chinese Academy of Agricultural Sciences, Beijing, China; 2 Qiyang Agro-ecosystem of National Field Experimental Station, Hunan, China; Old Dominion University, UNITED STATES

## Abstract

The effects of soil type and organic material quality on the microbial biomass and functional diversity of cropland soils were studied in a transplant experiment in the same climate during a 1-year field experiment. Six organic materials (WS: wheat straw, CS: corn straw, WR: wheat root, CR: corn root, PM: pig manure, CM: cattle manure), and three contrasting soils (Ferralic Cambisol, Calcaric Cambisol and Luvic Phaeozem) were chosen. At two time points (at the end of the 1st and 12th months), soil microbial biomass carbon (C) and nitrogen (N) (MBC and MBN) and Biolog Ecoplate substrate use patterns were determined, and the average well color development and the microbial functional diversity indices (Shannon, Simpson and McIntosh indices) were calculated. Organic material quality explained 29.5–50.9% of the variance in MBC and MBN when compared with the minor role of soil type (1.4–9.3%) at the end of the 1st and 12th months, and C/N ratio and total N of organic material were the main parameters. Soil properties, e.g., organic C and clay content were the predominant influence on microbial functional diversity in particular at the end of the 12th month (61.8–82.8% of the variance explained). The treatments of WS and CS significantly improved the MBC and microbial functional diversity indices over the control in the three soils in both sampling periods (*P* < 0.05). These results suggest that the application of crop straw is a long-term effective measure to increase microbial biomass, and can further induce the changes of soil properties to regulate soil microbial community.

## Introduction

Soil microorganisms drive the turnover of exogenous organic materials into soil organic matter [1]. The quality of applied organic materials can regulate microbial abundance and function [2–4]. In China, large amounts of agricultural byproducts are produced because high agricultural productivity is being pursued to meet the food demands of the huge population. In China in 2011, 863 million tons of crop straw and 3 trillion tons of livestock manure were produced [5–6]. These excessive byproducts have created a series of negative environmental effects, such as atmospheric pollution, water eutrophication and so on. In agricultural systems, the return of organic materials to the soil is the most prevalent practice to maintain or improve soil fertility. However, the size and function of microorganisms is different in different soils, for example, high content of soil organic matter is generally associated with high microbial abundance and diversity [7–8]. Increased understanding of the size and function of microorganisms after application of different organic materials in different types of agricultural soil is therefore helpful to clarify the effect of organic materials and soil types on microbial characteristics.

Soil microbial biomass carbon (C) and nitrogen (N) (MBC and MBN) reflect microbial size and soil fertility status, and they act as the living nutrient pool in soil [9]. Soil microbial functional diversity is linked with the stability of soil microbial communities and levels of soil biodiversity [10]. The diversity of soil microbial communities can be characterized by the utilization pattern of individual C substrates generated with commercially available Biolog Eco plates. These community-level physiological profiles (CLPPs) have provided a rapid means for evaluating the structure and species composition of soil microbial communities. The average well color development (AWCD) and the functional diversity indices, including Shannon (*H’*), Simpson (*D*) and McIntosh (*U*) indices, are important diagnostic indicators of soil quality [11]. Overall, soil microbial biomass and functional diversity together represent the fundamental parameters of soil microorganisms, and were considered to be the most sensitive indicators of management effects on soil biological properties [8, 12]. The analysis of soil microbial characteristics can indicate the status of soil fertility and ecosystem function.

The quality of organic materials affects the microbial biomass and community structure [13–16]. Microbial biomass carbon and Shannon’s diversity index after amendment with labile organic materials with low lignin content were significantly higher than that after amendment with recalcitrant organic materials with high lignin content [16–17]. In agricultural systems, the available organic materials generally include crop residues and livestock manures; crop residues are characterized by higher C:N ratio and lower available nutrient content in comparison with manure [15]. Generally, the microbial biomass or functional diversity after amendment with crop straws was lower than that with manure in agricultural soils because of the low availability of C sources and nutrients in crop residues [13, 15–18]. To date, most studies of soil microbial characteristics with different organic materials amendment concentrated mainly on a certain soil or different soils under controlled laboratory conditions [17, 19]; little information is reported about comparative studies of microbial characteristics dynamics in soils developed from different parent materials after amendment with different organic materials under field conditions.

Soil properties, such as parent material, soil organic matter, pH and clay content can also influence soil microbial biomass and functional diversity [19–21]. Soil parent material provides the basic nutritional environment for development of the microbial community [22–23], and during soil formation the soil microbial communities can be changed [19, 21]. Soil organic matter provides energy to microbes, and soil with higher content of SOM generally has higher microbial biomass and functional diversity [7, 17, 24–26]. Soil pH plays an important role in shaping microbial community composition [27–30]; soil pH was negatively correlated with soil biomass and positively correlated with AWCD [11, 13]. Soil texture can also affect the soil nutrient status and water content, thus affecting the living environment and metabolic activity of microorganisms [31–32]. Ranjard and Richaume (2001) [33] found that 40–70% of the bacteria were located in the 2–20 and < 2 μm aggregates. Consequently, the comparison of microbial characteristics in different soil types can improve our understanding of the influence of soil properties on microbes.

In China, the Ferralic Cambisol, Calcaric Cambisol and Luvic Phaeozem are the typical intensive cropland soils. Currently, these Chinese cropland soils have the obvious trend of acidification because of excessive N fertilizer application when compared with those soils 30 years ago [34]. Ferralic Cambisol is found in the subtropical region with an acidic soil environment [35], Calcaric Cambisol is found in the warm temperate region with a weak basic or neutral soil environment, and Calcaric Cambiso is found in the cold temperate region with a weak acidic or neutral soil environment. To better compare the effects of exogenous organic materials and soil type on the microbial characteristics and to eliminate the effect of climate factors, Calcaric Cambisol and Luvic Phaeozem were moved to the subtropical region to accentuate the effects of global warming and soil acidification. The objectives of the present study were therefore (1) to explore the changes in microbial biomass and functional diversity during the decomposition of organic materials in different soil types, and (2) to quantify the contributions of soil type and quality of organic materials to microbial biomass and functional diversity.

## Materials and methods

### Soils collection

**Fig 1:**
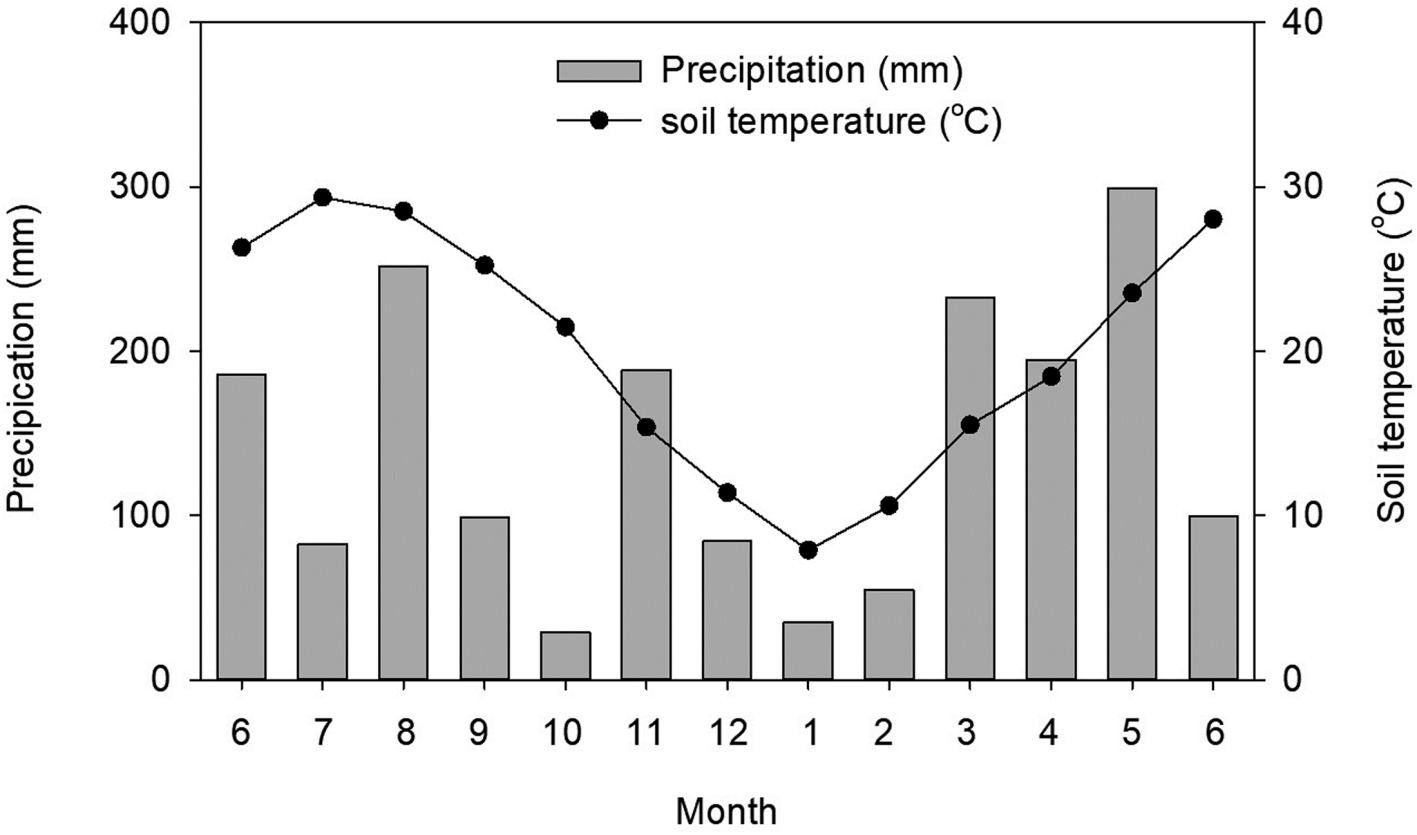
Precipitation and soil temperature from June 2012 to June 2013 at Qiyang Station.

Three typical cropland soils including Ferralic Cambisol, Calcaric Cambisol and Luvic Phaeozem (FAO classification) were collected from the national long-term monitoring stations of soil fertility which were established by Qiyang Agro-ecosystem of National Field Experimental Station, Henan Academy of Agricultural Sciences and Jilin Academy of Agricultural Sciences, respectively. The Ferralic Cambisol developed from the quaternary red soil was located in Qiyang County, Hunan Province; this region has a subtropical climate, with an annual average temperature of 18°C and an average annual rainfall of 1255 mm. The Calcaric Cambisol developed from alluvial sediments of the Yellow River was located in Yuanyang County, Henan Province; this region has a temperate sub-humid climate, with an annual average temperature of 14.5°C and an average annual rainfall of 450–600 mm. The Luvic Phaeozem derived from the quaternary loess sediments was located in Gongzhuling County, Jilin Province; this region is characterized by temperate sub-humid climate, with an annual average temperature of 4–5°C and an average annual rainfall of 450–600 mm. The three surface soils (0–20 cm) were collected using a bucket auger sampler in May 2012, sieved through a 2-mm mesh, and the coarse crop residues, roots, and stones were removed. The soil physicochemical properties were shown in Table 1 and the average monthly rainfall and temperature of the study site during the sampling period were shown in Fig 1.

**Table 1:** Basic physicochemical properties of three soils.

Properties	Ferralic Cambisol	Calcaric Cambisol	Luvic Phaeozem
Location	111°52′N, 26°45′E	113°41′N, 35°00′E	124°48′N, 43°30′E
Organic carbon (g kg^-1^)	10.1±0.1b	6.3±0.1c	15.6±0.2a
Total N (g kg^-1^)	1.2±0.1a	0.7±0.1b	1.4±0.2a
Total P (g kg^-1^)	0.9±0.1a	0.7±0.1b	0.6±0.1c
Total K (g kg^-1^)	12.8±0.1b	21.3±0.3a	22.9±0.3a
pH (soil:water = 1:2.5)	5.2±0.1b	8.3±0.2a	5.9±0.1b
Sand (%)	18.8±0.3c	67.1±1.1a	38.7±0.6b
Silt (%)	31.9±0.4a	18.7±0.2c	28.7±0.4b
Clay (%)	43.9±0.8a	10.1±0.2c	29.3±0.5b

Values are mean ± standard error of three replicates. Different letters in the same row indicate significant difference at *P*<0.05 among three soils.

### Preparation of organic material

Six kinds of organic materials were chosen, including wheat (*Triticum aestivum* L.) straw (WS), corn (*Zea mays* L.) straw (CS), wheat root (WR), corn root (CR), pig manure (PM), and cattle manure (CM). All the organic materials were oven-dried at 60°C, and passed through a 2-mm sieve. The chemical characteristics of these organic materials were shown in Table 2.

**Table 2:** Chemical characteristics of wheat straw (WS), corn straw (CS), wheat root (WR), corn root (CR), pig manure (PM), and cattle manure (CM).

Organic materials	Total C (g kg^-1^)	Total N (g kg^-1^)	C: N ratio	Cellulose (%)	Hemicellulose (%)	Lignin (%)
Wheat straw (WS)	450.7±16a	5.1±0.8c	88.0±5.1a	36.4±0.0b	30.6±0.2b	7.8±0.1c
Corn straw (CS)	461.8±14a	5.2±0.6c	89.0±4.2a	36.5±0.2b	33.2±0.0a	4.0±0.0e
Wheat root (WR)	329.7±17d	3.7±0.3d	89.6±4.3a	28.2±1.6c	25.0±1.8c	6.4±0.3d
Corn root (CR)	473.3±15a	5.8±0.5c	81.5±3.8a	40.2±0.2a	33.2±0.2a	6.4±0.3d
Pig manure (PM)	358.0±11c	11.6±0.8b	31.0±3.1b	28.0±0.2c	23.8±0.1c	12.1±0.0a
Cattle manure (CM)	396.9±12b	18.0±0.9a	22.0±2.8b	19.7±0.1d	25.6±0.4c	8.6±0.2b

Values are mean ± standard error of three replicates. Different letters in the same column indicate significant difference at *P*<0.05 among different materials.

### Experimental design

The experiment was carried out from June 5, 2012 to June 5, 2013 in the long-term experimental station of the Chinese Academy of Agricultural Sciences, Qiyang County (111°52′32″N, 26°45′12″E), Hunan Province. Before the experiment, both Calcaric Cambisol from Zhengzhou and Luvic Phaeozem from Gongzhuling were moved to Qiyang County.

Soil type was the main plot factor and organic material was the subplot factor in a split-plot design with six replicates; three replicates were sampled at the end of the 1st month, and the other three replicates were sampled at the end of the 12th month. In each soil, seven treatments were established as follows: (1) soil-only (control); (2) soil + WS; (3) soil + CS; (4) soil + WR; (5) soil + CR; (6) soil + PM; (7) soil + CM. A total of 126 nylon bags (20 × 15 cm^2^, 0.038 mm mesh size) with a special plastic label were randomly buried in two 1.5 × 1.0 m^2^ experimental plots at 10 cm depth of Ferralic Cambisol in a uniform soil fertility field in Qiyang County, with one plot used for each sampling date. In each bag, 200 g (oven-dried basis) experimental soil (Ferralic Cambisol, Calcaric Cambisol, Luvic Phaeozem) was thoroughly mixed with organic material at a ratio of 15 g C kg^−1^ soil, which was equivalent to 34 t C ha^−1^ returned to the soil. The amount of different organic materials in each bag was described in S1 Table. During the experimental period, no crops were planted in the plots, and weeds were removed regularly by hand to decrease the effect of weed roots on the nylon bags. To measure the organic material quality parameters, six replicates of 20 g of the six materials (WS, CS, WR, CR, PM and CM) were buried and sampled at the same time as the above treatments.

### Sampling and analysis

On the sampling day, each bag was weighed after the attached soil on the outer wall of the bag was carefully removed. Part of the fresh soil in the bag was taken to determine microbial CLPPs, MBC and MBN, while the remaining soil was air-dried to determine soil organic C (SOC), total N, and pH. Total fiber content of organic material (cellulose, hemicellulose and lignin), total organic C, and total organic N were determined.

### BIOLOG analysis

Microbial CLPPs in soil were determined by Biolog Eco plates (Biolog, Hayward, CA, USA). Briefly, 5 g of fresh soil was shaken in 45 ml of sterile saline solution (0.85% NaCl w/v) for 30 min at the rate of 180 rpm, and then the mixture was diluted 100-fold. Aliquots of 150 μl of the 10^−3^ suspension was incubated in each well of Biolog Eco plates at 28°C and the absorbance was measured at 590 nm with an Emax precision microplate reader (Biolog, Hayward, CA, USA). The readings at 96 h incubation collected by Microlog Rel. 4.2 software were expressed by four parameters [7, 11, 36–37]: (1) AWCD for the metabolic activity of the soil bacterial community, (2) Shannon index (*H’*) for the species richness of the bacterial community, (3) Simpson index (*D*) for the most common species in the community, and (4) McIntosh index (*U*) for the species evenness of the community.

The AWCD is calculated to reflect the utilization of single C sources by soil microorganisms:

AWCD=∑(Ci-R)/31

where *Ci* is the absorbance of well i and R is the absorbance of the control well. When *Ci—R* <0 or *Ci—R* <0.06, the values were set to 0 [38].

The functional diversity indices were calculated as described by Zhong et al. [11]:

H'=-∑Pi∙ln(Pi)

$$D\;=\;1-\sum {\left(Pi\right)}^{2}$$

$$U\;=\;\sqrt{\sum {ni}^{2}}$$

where *Pi* is the ratio of activities on each substrate to the sum of activities on all substrates and *ni* is the activities on each substrate.

### Microbial biomass

Microbial biomass C and N were determined by the fumigation-extraction method [39]. A 20-g subsample of soil (oven-dried basis) was fumigated by exposing the soil to alcohol-free CHCl_3_ vapor in a sealed vacuum desiccator for 24 h. The fumigated soil was evacuated repeatedly in a clean empty desiccator until the odor of CHCl_3_ was not detected, and then extracted with 80 ml 0.5 M K_2_SO_4_ (soil:K_2_SO_4_ = 1:4) for 30 min. The extraction of non-fumigated soil was the same as that of the fumigated soil. Microbial biomass C and N were estimated by the difference between the total organic C or total N in the fumigated and non-fumigated extracts with a conversion factor (K_EC_) of 0.38 and (K_EN_) of 0.45 [40–41], respectively.

### Physicochemical analysis of soil and organic material

Soils and organic materials were analyzed for organic C and total C by dichromate oxidation and total N by Kjeldahl digestion. Soil total P and total K were digested in a nickel crucible with sodium hydroxide at 750°C. Soil available P was extracted with 0.5 M NaHCO_3_. Soil total P and available P were determined by the molybdenum-blue method at a wavelength of 880 nm. Soil available K was extracted with 1 M NH_4_OAc. Soil total K and available K were determined using atomic absorption spectrophotometry. Soil pH was determined in water (soil: water = 1: 2.5). Soil clay, silt and sand were determined by the pipette method. Total fiber content of organic material (cellulose, hemicellulose and lignin) was determined by the method described by van Soest [42].

### Statistical analysis

Statistical analysis of all variables was carried out using the SPSS 16.0 software package. To evaluate the primary factors influencing microbial parameters, we analyzed MBC, MBN, AWCD, *H’*, *D* and *U* using a two-way analysis of variance (ANOVA) with soil and organic material types as independent factors and permitted to interact. A one-way ANOVA was used to determine the differences of soil properties, chemical characteristics of organic material, and the above microbial parameters among organic material treatments at each soil. The differences among treatments with separation of means by Tukey’s HSD (α = 0.05) test at *P* < 0.05. Principal component analysis (PCA) of the Ecoplate data was performed to characterize the effect of different organic materials on soil microbial community functions, and the differences of the factor scores of the first principal component (PC1) axis among organic material treatments at each soil were tested using a one-way ANOVA by Tukey’s HSD (α = 0.05) test at *P* < 0.05. Stepwise multiple regression analysis was applied to determine the key factors influencing microbial properties.

## Results

### Microbial biomass carbon and nitrogen (MBC and MBN)

**Fig 2:**
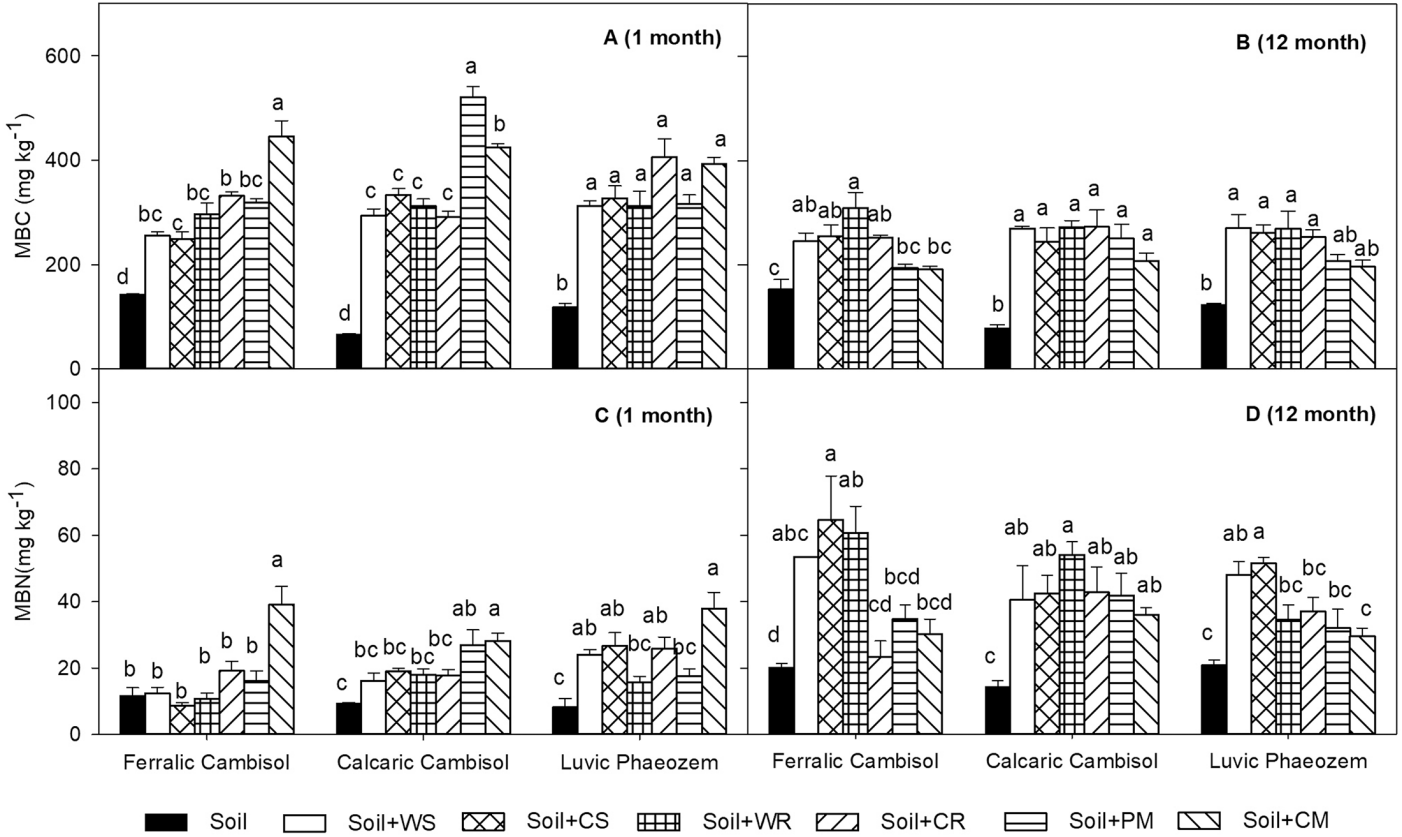
Soil microbial biomass carbon and nitrogen (SMBC, SMBN) in Ferralic Cambisol, Calcaric Cambisol, and Luvic Phaeozem at the end of 1st and 12th months with the amendment of different organic materials. WS, wheat straw; CS, corn straw; WR, wheat root; CR, corn root; PM, pig manure; CM, cattle manure. Different letters indicate significant differences at *P* < 0.05 among different materials in the same soil.

At the end of the 1st month, the contributions of soil type and organic material type were significant in explaining the variance in MBC and MBN, and explained 6.9 and 43.6% of the variance in MBC, as well as 9.3 and 50.9% of the variance in MBN, respectively (*P* < 0.05; Table 3). Significantly higher MBC and MBN were found in Calcaric Cambisol and Luvic Phaeozem than that in Ferralic Cambisol regardless of organic material type (*P* < 0.05, Fig 2A and 2C). When compared with the control, all organic material treatments significantly increased the MBC while only the CM and PM treatments significantly increased the MBN in the three soils (*P* < 0.05, Fig 2A and 2C).

**Table 3:** Analysis of variance to evaluate the two primary factors (soil type, organic material type) that influence soil microbial biomass carbon (MBC), soil microbial biomass nitrogen (MBN), average well color development (AWCD), Shannon index (*H’*), Simpson index (*D*), and McIntosh index (*U*) during the whole experimental period.

Parameters	Source of variation	df	%SS	F	*P*	%SS	F	*P*
		1 month				12 month		
**MBC**								
	Soil	2	6.9	9.9	<0.05	1.4	0.6	0.561
	Organic material	5	43.6	25.2	<0.05	45.3	7.5	<0.05
	Soil × Organic material	10	37.1	12.2	<0.05	9.7	0.8	0.630
	Residuals	36	12.5			43.6		
**MBN**								
	Soil	2	9.3	8.1	<0.05	3.0	1.5	0.245
	Organic material	5	50.9	17.9	<0.05	29.5	5.7	<0.05
	Soil × Organic material	10	19.3	3.4	<0.05	30.2	2.9	<0.05
	Residuals	36	20.5			37.4		
**AWCD**								
	Soil	2	24.8	181.3	<0.05	80.0	1226.0	<0.05
	Organic material	5	38.4	112.2	<0.05	5.4	111.9	<0.05
	Soil × Organic material	10	34.3	50.0	<0.05	13.3	60.1	<0.05
	Residuals	36	2.5			1.2		
**Shannonindex (*H’*)**								
	Soil	2	74.4	380.6	<0.05	65.7	178.5	<0.05
	Organic material	5	13.7	28.1	<0.05	11.1	12.1	<0.05
	Soil × Organic material	10	8.4	8.6	<0.05	16.6	9.0	<0.05
	Residuals	36	3.5			6.6		
**Simpson index (*D*)**								
	Soil	2	45.3	74.1	<0.05	61.8	122.8	<0.05
	Organic material	5	22.7	14.7	<0.05	10.1	8.0	<0.05
	Soil × Organic material	10	21.3	6.9	<0.05	19.1	7.6	<0.05
	Residuals	36	10.7			9.0		
**McIntosh index (U)**								
	Soil	2	34.2	250.1	<0.05	82.8	1348.5	<0.05
	Organic material	5	40.3	118.1	<0.05	5.5	35.8	<0.05
	Soil × Organic material	10	23.1	33.8	<0.05	10.6	34.6	<0.05
	Residuals	36	2.5			1.1		

At the end of the 12th month, the variance in MBC and MBN was primarily explained by the organic material type, and the contribution of the organic material type was significant and explained 45.3% of the variance in MBC and 29.5% of the variance in MBN (*P* < 0.05, Table 3). The WS, CS, WR and CR treatments significantly increased the MBC while only the WS and CS treatments significantly increased the MBN when compared with the control in the three soils (*P* < 0.05, Fig 2B and 2D). When compared with the end of the 1st month, the MBC at the end of the 12th month decreased by 21.5–28.7%, and the MBN at the end of the 12th month increased by 62.9–143.7% in the three soils (Fig 2).

### Metabolic activity and microbial functional diversity

**Fig 3:**
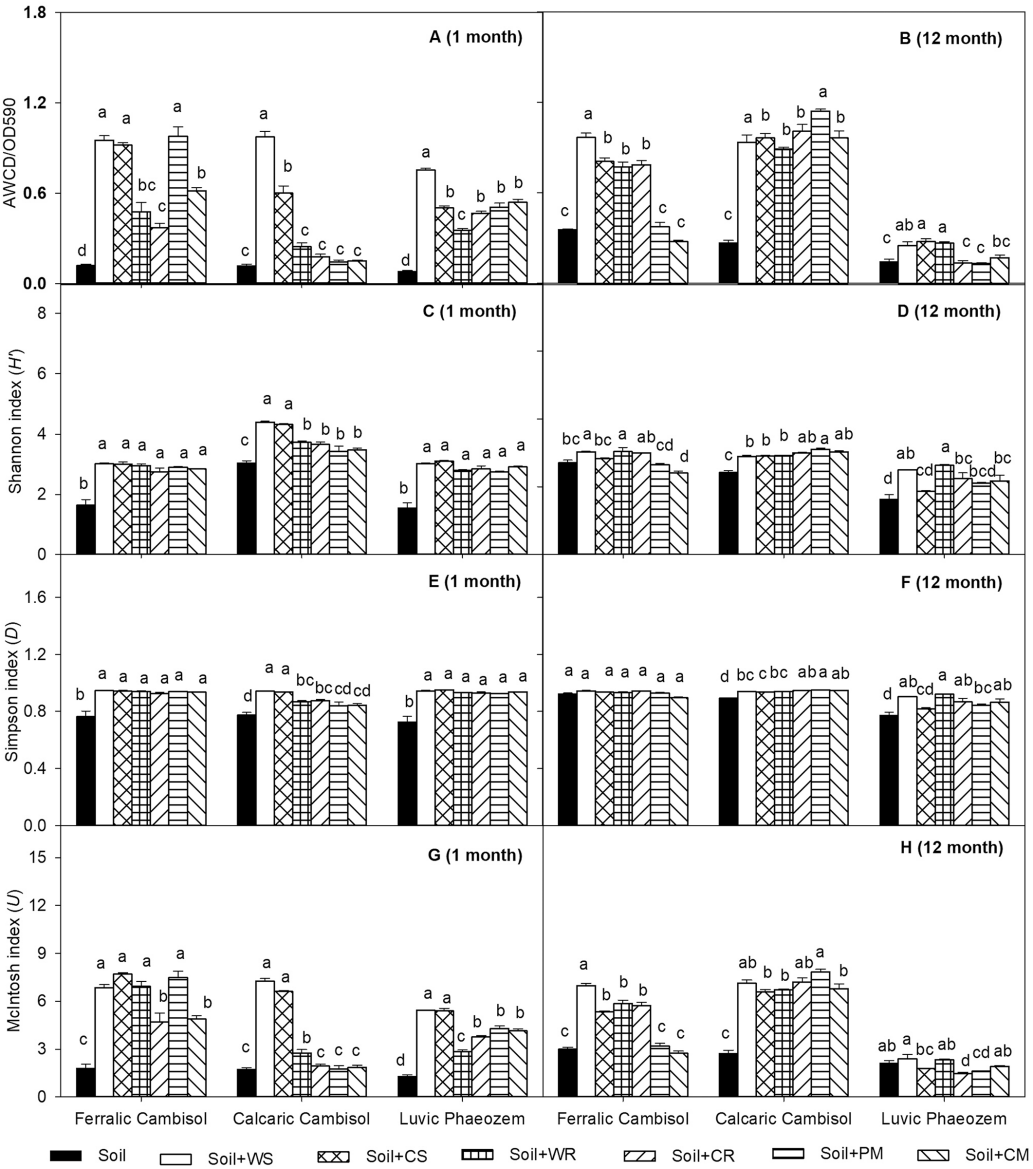
Average well color development (AWCD), Shannon index (*H’*), Simpson index (*D*) and McIntosh index (*U*) in Ferralic Cambisol, Calcaric Cambisol, and Luvic Phaeozem at the end of the 1st and 12th months with the amendment of different organic materials. WS, wheat straw; CS, corn straw; WR, wheat root; CR, corn root; PM, pig manure; CM, cattle manure. Different letters indicate significant differences at *P* < 0.05 among different materials in the same soil.

At the end of the 1st month, the contributions of soil type and organic material type were significant in explaining the variance in microbial functional diversity (*P* < 0.05, Table 3). The AWCD and McIntosh index was primarily explained by the organic material species (38.4 and 40.3%, respectively), and the Shannon and Simpson indices were primarily explained by soil type (74.4 and 45.3%, respectively). The microbial functional diversity of Ferralic Cambisol and Luvic Phaeozem in all organic material treatments was significantly increased when compared with the control (*P* < 0.05), while only the WS and CS treatments significantly increased all functional diversity indices in Calcaric Cambisol when compared with the control (*P* < 0.05, Fig 3A, 3C, 3E and 3G).

At the end of the 12th month, the contributions of soil type and organic material type were also significant in explaining the variance in the microbial functional diversity (*P* < 0.05, Table 3), with 61.8–82.8% of the variances in functional diversity primarily explained by soil type (*P* < 0.05, Table 3). The WS and WR treatments significantly increased the AWCD, Shannon and McIntosh indices in Ferralic Cambisol and Luvic Phaeozem when compared with the control, and all organic material treatments increased the functional diversity indices in Calcaric Cambisol when compared with the control (*P* < 0.05, Fig 3B, 3D, 3F and 3H).

### Carbon substrate utilization patterns of soil microbial communities

**Fig 4:**
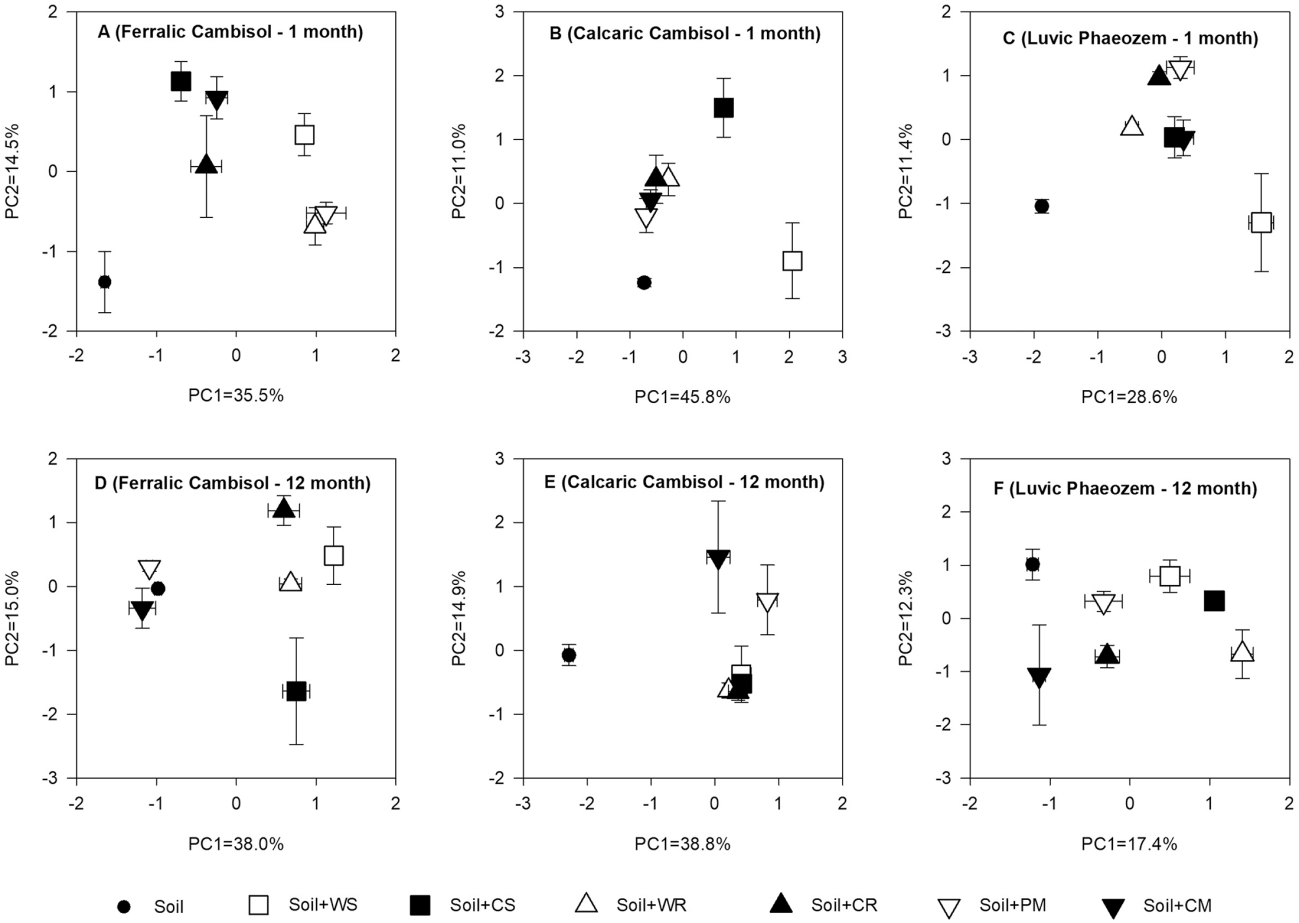
Principal component analysis of carbon sources utilization by different soil microbial communities in Ferralic Cambisol, Calcaric Cambisol, and Luvic Phaeozem at the end of the 1st and 12th months with the amendment of different organic materials. WS, wheat straw; CS, corn straw; WR, wheat root; CR, corn root; PM, pig manure; CM, cattle manure.

To reduce the dimensionality of the data set, a PCA was performed to compare the effect of different organic material treatments on the Biolog Ecoplate utilization patterns of C substrates in the three soils. At the end of the 1st month, the ANOVA for principal component 1 (PC1) indicated that the patterns of substrate utilization between the organic materials and the control treatments were significantly different in Ferralic Cambisol and Luvic Phaeozem (*P* < 0.05), and that they were significantly different between the WS and CS treatments and the control in Calcaric Cambisol (*P* < 0.05, Fig 4A–4C). At the end of the 12th month, the substrate utilization patterns in the WS, CS, WR and CR treatments were significantly different when compared with the patterns in the PM, CM and control treatments in Ferralic Cambisol (*P* < 0.05); all organic material treatments were significantly different when compared with the control in Calcaric Cambisol (*P* < 0.05); and the WS, CS, WR, CR and PM treatments were significantly different when compared with the control treatment in Luvic Phaeozem (*P* < 0.05, Fig 4D–4F).

A high Pearson correlation coefficient (> 0.6) for PC1 in the organic material treatments was shown in Table 4. At the end of the 1st month, the C substrate use pattern was primarily associated with increased utilization of carbohydrates, amino acids and polymer in Ferralic Cambisol; carbohydrates, amino acids, carboxylic acid, polymer and amine in Calcaric Cambisol; carbohydrates, carboxylic acids, amino acid and polymer in Luvic Phaeozem. At the end of the 12th month, the C substrate use pattern was changed in the three soils. It was associated with increased utilization of carbohydrates, amino acids and amine in Ferralic Cambisol and Calcaric Cambisol, and carbohydrates, amino acids, carboxylic acids and polymer in Luvic Phaeozem.

**Table 4:** Substrates with significant Pearson’s correlation coefficients (r > 0.6) for principal component 1 in the principal component analysis of substrate utilization patterns of the soil microbial community for three soils during the whole experiment.

Type	Substrate name	Ferralic Cambisol	Calcaric Cambisol	Luvic Phaeozem	Ferralic Cambisol	Calcaric Cambisol	Luvic Phaeozem
		1 month			12month		
**Carbohydrate**	β-methyl-D-Glucoside	0.880	0.880		0.949	0.915	
	ᴅ-Galactonic acid lactone	0.736	0.847	0.764	0.668	0.945	0.735
	ᴅ-Xylose						
	ᴅ-Galacturonicacid	0.888	0.912	0.608	0.966	0.949	
	γ-Lactone						
	i-Erythritol			0.693	0.630		
	ᴅ-Mannitol	0.824	0.932	0.833	0.883	0.931	
	*N*-acetyl-ᴅ-Glucosamine	0.864	0.885	0.680	0.945	0.929	
	ᴅ-Glucosaminic acid						0.783
	ᴅ-cellobiose	0.911	0.735				
	α-ᴅ-Glucose-1-phosphate	0.877	0.897		0.953	0.812	
	α-ᴅ-lactose	0.782	0.900		0.809	0.864	
	ᴅ,L-α-Glyce	0.767	0.849			0.803	
**Amino acid**	L-Arginine			0.602			
	L-Asparagine	0.837	0.934		0.686	0.939	
	L-Phenylalanine	0.724	0.713			0.741	0.711
	L-Serine	0.871	0.906		0.946	0.882	
	L-threonine					0.766	0.733
	Glycyl-L-glutamic	0.740	0.899		0.918	0.752	
**Carboxylic acids**	Pyruvic acid methyl ester						
	γ-Hydroxybutyric acid		0.750	0.946			
	Itaconic acid						
	α-Ketobutyric acid						
	ᴅ-Malic acid			0.714			0.686
**Polymer**	Tween 40		0.617				
	Tween 80			0.703			0.687
	α-Cyclodextrin						
	Glycogen	0.681					
**Phenolic compounds**	2-Hydroxy benzoic Acid						
	4-Hdroxy benzoic acid						
**Amine**	Phenylethyl-anime						
	Putrescine		0.853		0.729	0.771	

### The relationships among microbial properties, organic material quality and soil physicochemical properties

The C/N ratio and N content of organic materials significantly affected the MBC and MBN at the end of the 1st and 12th months (*P* < 0.05), and soil clay significantly affected MBC at the end of the 1st month (*P* < 0.05, Table 5). At the end of the 1st month, soil clay content significantly influenced AWCD and *U*, pH significantly influenced *D*, and total nitrogen significantly influenced *H’* (*P* < 0.05). The lignin content of organic materials significantly influenced *H’* and *D* at the end of the 1st month (*P* < 0.05). At the end of the 12th month, the soil organic C (SOC) and C/N ratio of organic materials significantly influenced AWCD, *H’*, *D* and *U* (*P* < 0.05), and the clay content significantly influenced *H’* and *D* (*P* < 0.05).

**Table 5:** Regressions of organic material quality variables and soil properties with microbial parameters (*n* = 54) at the end of the 1st and 12th months with the amendment of different organic materials (*P* < 0.05).

Regression equation	*R*^2^
**1 month**	
MBC = 467.895–1.526 C/N—1.347 clay	0.38
MBN = 13.087 + 67.799 OTN	0.44
AWCD = 0.267 + 0.010 clay	0.24
Shannon index (*H’*) = 5.038–2.801 STN + 0.127 SOC– 0.200 lignin	0.78
Simpson index (*D*) = 1.057–0.018 pH– 0.021 lignin	0.48
McIntosh index (*U*) = 3.300 + 0.078 clay—5.653 OTN	0.36
**12 month**	
MBC = 154.602 + 3.788 C/N	0.43
MBN = 21.122 + 0.870 C/N	0.22
AWCD = 1.105–0.081 SOC+ 0.010 C/N	0.84
Shannon index (*H’*) = 2.921–0.077 SOC + 0.014 C/N + 0.006 clay	0.73
Simpson index (*D*) = 0.938–0.007 SOC + 0.001 C/N + 0.001 clay	0.68
McIntosh index (*U*) = 7.769–0.524 SOC + 0.073 C/N	0.86

Soil properties: SOC, soil organic carbon; STN, soil total nitrogen; clay; pH; Organic material quality: OTN, total nitrogen; C/N ratio; lignin.

## Discussion

### Effects of soil properties and organic materials quality on microbial biomass

Soil microbial biomass represents the amount of microbes in soil, and was successfully used to detect short-term changes in soil functioning to predict organic C accumulation in soil under organic management [20]. The quality of organic material, e.g., the C availability, the C/N ratio and N content, determines the size of the microbial biomass [13, 43–45] (Table 3). Carbon sources can provide energy for microorganisms [46–47], and microorganisms can grow rapidly when they encounter abundant C sources, e.g., the significant increase in MBC in organic materials amendment treatments when compared with the control treatment in the three soils at the end of the 1st month (Fig 2). The C/N ratio of organic materials has generally been shown to be a good predictor of the decomposition of organic materials [45, 48], and organic materials with low C/N ratio can supply sufficient nutrients for microbes [49–50], which was shown by the significantly higher MBC and MBN in manure treatments than those in crop residue treatment at the end of the 1st month (Table 2, Fig 2). Nevertheless, at the end of the 12th month (Fig 2) the crop residue amendments with high C/N ratio induced significantly higher MBC and MBN than the manure amendments. Generally, soil N immobilization occurred with organic materials amendment [47, 51]. As the experiment proceeded, the amount of available C and N sources decreased and further entered the environment, e.g., as C and N gaseous emissions, dissolved organic C and nitrate leaching under the high precipitation in the experimental subtropical region, especially in the manure amendment treatments; large amounts of easily decomposable and passive decomposable C sources and nutrients were activated by microbial metabolism, and then these activated C sources and nutrients can be easily lost. Conversely, the N limitation was more serious in crop material treatments with high C/N ratio than in manure treatments (S1 Fig) [46]; the immobilized N induced by crop materials can be recycled in microorganisms with crop materials decomposition [47, 52]. A ^15^N-tracer experiment also demonstrated that organic materials with high C/N ratio prolong nutrient retention in soil through microbial metabolism [47]. The calculated ratio of MBC to MBN between the 1st and 12th months in this study supports the above phenomenon.

Soil properties had less influence on microbial biomass when compared with the organic material quality, with significant effects only observed at the end of the 1st month (Table 3). In the present study, organic materials amendment might have completely obscured the effect of soil properties on microbial biomass. Generally, soils with high SOC content had high microbial biomass [25–26, 53–54], and nutritional stress might occur when SOC was less than 1% [53]. When compared with the other two soils in our study (Table 1), Luvic Phaeozem had high microbial biomass because SOC in Luvic Phaeozem is 1.5–2.5 times that of the other two soils. Further, stepwise multiple regression analysis showed that clay content was negatively correlated with MBC after the addition of organic materials at the end of the 1st month. Müller et al. [55] reported that the clay protective effect of nutrients on microbial biomass was limited, and the increase in clay content could not improve the response of microorganisms to organic material amendment when clay content was > 25%; for example, Ferralic Cambisol had higher clay content than the other two soils in the present study. Meanwhile, the low pH in the Ferralic Cambisol (pH = 5.2) would reduce the utilization of labile substrate by soil microbes [56–57] because of the toxic exchangeable Al in low pH soil [58]; however, the integrated effect of SOC, clay content and organic materials amendment could affect the response of microbial biomass to pH as shown by the non-significance of pH in explaining microbial biomass in the stepwise multiple regression analysis.

### Effects of soil properties and organic materials quality on microbial functional diversity

The average well color development (AWCD) and the functional diversity indices including Shannon, Simpson and McIntosh indices were often used to investigate the general structure and functional potential of soil microbial communities [13, 24]. The integrated effect of soil type and organic material amendment significantly (*P* < 0.05) affected the microbial functional diversity. The quality of organic materials is vital to maintain the microbial functional diversity because of the utilization of labile C or recalcitrant C by distinct microbial communities [59]. Lignin is resistant to biodegradation and higher lignin content depresses microbial metabolism; this resulted in the negative correlation between lignin content and the diversity indices (Shannon and Simpson indices) in different organic material treatments at the end of the 1st month [17, 45]. At the end of the 12th month in the present study, the microbial functional diversity indices were positively correlated with C/N ratio of organic materials (Table 5), and the microbial communities in crop residue treatments were separated from those in the control treatment in the three soils (Fig 4); this is because the decomposable C sources from crop residue, including cellulose and hemicellulose, and the lower lignin content in crop residues when compared with that in manures supported high microbial functional diversity [17, 44, 60].

Soil properties were more important than organic material properties in explaining the microbial functional diversity as shown in Table 3 [13, 18–19, 24]. At the end of the 1st month, the increase in AWCD and McIntosh index with increased clay content was because silt and clay particles generally supported larger and more diverse microbial communities than sand particles [61]. High soil N content negatively affected soil microbial communities and led to a decrease in the microbial functional diversity by altering the supply and quality of organic matter [27, 62]; which resulted in significantly lower Shannon index in Ferralic Cambisol and Luvic Phaeozem than that in Calcaric Cambisol. Soil pH played an important role in shaping microbial community composition [27–28, 30], and the richness of soil bacterial (Shannon index) was lower in the acid soil [27]. The present study was not all consistent with the previous reports, although the Shannon index in Ferralic Cambisol and Luvic Phaeozem with lower initial pH (Table 1) was lower than that in Calcaric Cambisol at the end of the 1st month (Fig 3C). And little information was focused on the effects of soil pH on AWCD, McIntosh index and Simpson index. The high precipitation in the study site would leach the soluble acid ions into the litter bags, thus limiting organic matter availability and inhibiting microbial metabolism (Fig 1) [63–64]. As a result, AWCD and McIntosh index were low in Calcaric Cambisol because of its high initial pH (Table 1) at the end of the 1st month. As the experiment proceeded, the soil microbial community in Calcaric Cambisol adapted to the experimental environment, and the low nutrient content in Calcaric Cambisol may encourage the microbes to assimilate exogenous C resources from the added organic materials [13, 65]; hence, significantly higher microbial functional diversity indices were found in Calcaric Cambisol at the end of the 12th month when compared with those in the 1st month. When compared with the other two soils, Luvic Phaeozem soil had the highest SOC content (Table 1) and significantly lower functional diversity indices at both sampling dates (Fig 3), and the reasons were that (1) Luvic Phaeozem *per se* had the lowest functional diversity as shown the control treatment (Fig 3), (2) soil with high organic matter has sufficient available C sources for microbial assimilation, and showed reduced assimilation of exogenous C sources by microbes when compared with the Ferralic Cambisol and Calcaric Cambisol with lower organic matter content [13, 65]. In addition, soil microbial communities were largely affected by historical factors such as geographic location and soil type due to microbes dwelling in soil [20, 23, 66–67]. It has been showed that soil microbial diversity decreased with the increase of latitude and was positively correlated with air temperature [68], and Luvic Phaeozem in this study was developed from the highest latitude and the annual average temperature (4–5°C) in its local region was lower than the other two soils. Hence, it explained the lower functional diversity in Luvic Phaeozem than the other two soils. Though, Luvic Phaeozem soil transfered from the temperate sub-humid region to the subtropical region, however, the short term effect of climate in this study (≤ 1 year) was not enough to alter the initial microbial communities because it had been reported that no significant responses of climate change on microbial communities within less than 10 years [69].

## Conclusions

Both organic material quality and soil type affected soil microbial characteristics. Organic material quality played a predominant role in controlling the microbial biomass at both sampling periods, and the main parameters of organic matter were C/N ratio and N content. Although manures, with low C/N ratio and high nitrogen content, significantly increased microbial biomass when compared with crop residues at the end of the 1st month (*P* < 0.05), the crop residues significantly increased the microbial biomass when compared with manures at the end of the 12th month (*P* < 0.05). After the easily available C was exhausted, soil properties regulated the microbial functional diversity, and the main parameters of soil properties were soil organic C and clay content. When compared with the manures, crop residues, in particular straws with low lignin and high C/N ratio, significantly increased the functional diversity indices at both sampling periods (*P* < 0.05). This study suggests that the application of straw is a long-term effective measure to increase microbial biomass, and can further induce the changes of soil properties to regulate soil microbial community.

## Supporting information

S1 Fig Nitrogen remaining in Ferralic Cambisol, Calcaric Cambisol, and Luvic Phaeozem with the amendment of different organic materials. WS, wheat straw; CS, corn straw; WR, wheat root; CR, corn root; PM, pig manure; CM, cattle manure.
(TIF)

S1 Table Amount of different organic materials in each nylon bag (on the basis of 100:1.5, soil: Added organic carbon ratio). (DOCX)
