# Supplementary figures and images for: Factors affecting soil microbial biomass and functional diversity with the application of organic amendments in three contrasting cropland soils during a field experiment
Source: PLoS One. 2018 Sep 13;13(9):e0203812. doi: 10.1371/journal.pone.0203812 (PMC6136761; doi:10.1371/journal.pone.0203812)

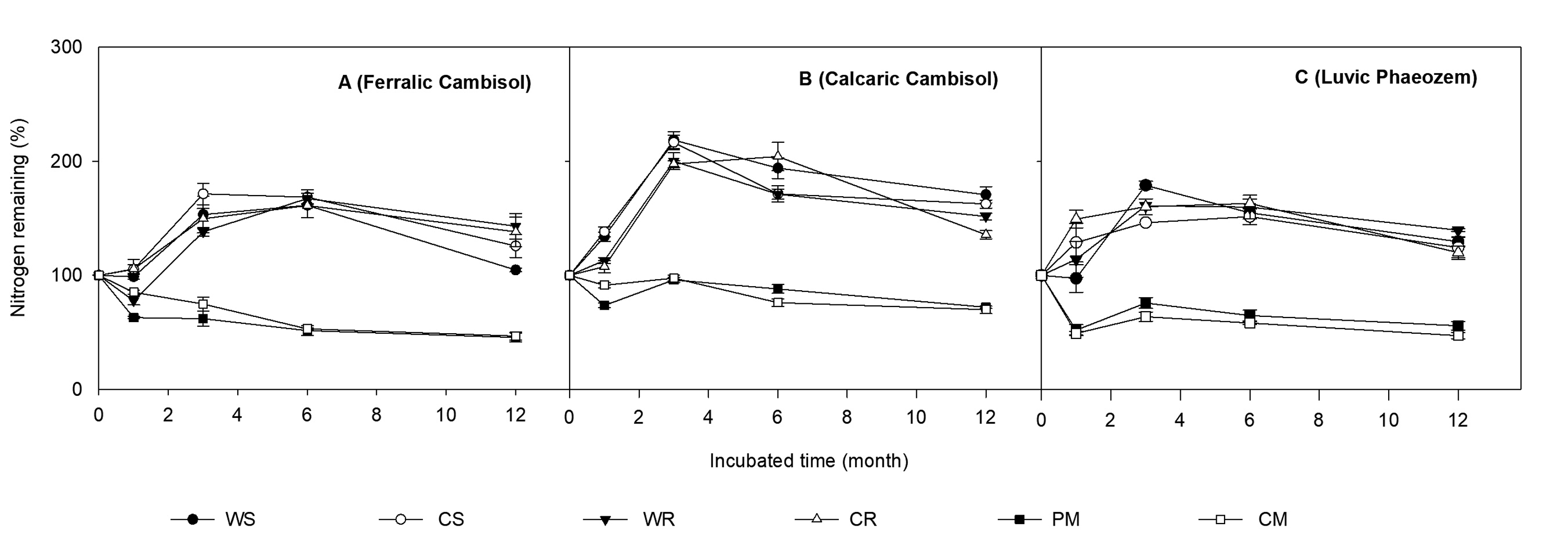

Supplement: S1 Fig — WS, wheat straw; CS, corn straw; WR, wheat root; CR, corn root; PM, pig manure; CM, cattle manure. (TIF) [file pone.0203812.s001.tif]
