# Supplementary material for: Factors affecting soil microbial biomass and functional diversity with the application of organic amendments in three contrasting cropland soils during a field experiment
Source: PLoS One. 2018 Sep 13;13(9):e0203812. doi: 10.1371/journal.pone.0203812 (PMC6136761; doi:10.1371/journal.pone.0203812)
Supplement: S1 Table — (DOCX) [file pone.0203812.s002.docx]

**S1 Table Amount of different organic materials in each nylon bag (on the basis of 100:1.5, soil: added organic carbon ratio).**

| Treatment | C concentration of organic material  (g kg^-1^) | Amount of organic material (g) | Amount of added organic carbon (g) | Amount of soil (g) |
| --- | --- | --- | --- | --- |
| Control | 0 | 0 | 0 | 200.00 |
| Soil+WS | 450.7 | 6.66 | 3.00 | 200.00 |
| Soil+CS | 461.8 | 6.50 | 3.00 | 200.00 |
| Soil+WR | 329.7 | 9.10 | 3.00 | 200.00 |
| Soil+CR | 473.3 | 6.34 | 3.00 | 200.00 |
| Soil+PM | 358.0 | 8.38 | 3.00 | 200.00 |
| Soil+CM | 396.9 | 7.56 | 3.00 | 200.00 |

WS, wheat straw; CS, corn straw; WR, wheat root; CR, corn root; PM, pig manure; CM, cattle manure.
